# Integrating the Capsule-like Smart Aggregate-Based EMI Technique with Deep Learning for Stress Assessment in Concrete

**DOI:** 10.3390/s24144738

**Published:** 2024-07-21

**Authors:** Quoc-Bao Ta, Quang-Quang Pham, Ngoc-Lan Pham, Jeong-Tae Kim

**Affiliations:** 1 Department of Ocean Engineering, Pukyong National University, 45 Yongso-ro, Nam-gu, Busan 48513, Republic of Korea; tabao838@pukyong.ac.kr (Q.-B.T.); bkdn06x3a@gmail.com (Q.-Q.P.); pnlan@pukyong.ac.kr (N.-L.P.); 2 Bridge and Road Department, Danang Architecture University, Danang 550000, Vietnam

**Keywords:** electromechanical impedance method, PZT sensor, smart aggregate, convolutional neural network, concrete structure, stress and damage monitoring

## Abstract

This study presents a concrete stress monitoring method utilizing 1D CNN deep learning of raw electromechanical impedance (EMI) signals measured with a capsule-like smart aggregate (CSA) sensor. Firstly, the CSA-based EMI measurement technique is presented by depicting a prototype of the CSA sensor and a 2 degrees of freedom (2 DOFs) EMI model for the CSA sensor embedded in a concrete cylinder. Secondly, the 1D CNN deep regression model is designed to adapt raw EMI responses from the CSA sensor for estimating concrete stresses. Thirdly, a CSA-embedded cylindrical concrete structure is experimented with to acquire EMI responses under various compressive loading levels. Finally, the feasibility and robustness of the 1D CNN model are evaluated for noise-contaminated EMI data and untrained stress EMI cases.

## 1. Introduction

Concrete structures may suffer local damage or deterioration over time; thus, it is crucial to routinely monitor stress variation and damage status to mitigate potential failures [1,2,3]. Various non-destructive testing techniques have been extensively utilized to monitor the integrity of concrete structures [4,5,6]. Among these, the electromechanical impedance (EMI) method has gained significant traction due to cost efficiency, reliable performance, and suitability for real-time monitoring [7]. The technique employs PZT transducers to extract damage information on the specific region of an investigated structure [8]. For damage monitoring, changes in EMI responses are estimated by recognizing changes in statistical patterns or observing shifts of impedance frequencies and magnitudes. 

For concrete health monitoring, the EMI technique with different sensing strategies has been developed to enhance the technique’s performance in assessing concrete damage [9,10,11,12,13,14]. Song et al. [10] proposed a smart aggregate (SA)-based EMI technique to mitigate the impact of noisy environments and temperature fluctuations on PZT sensors. Kong et al. [11,12] developed an embedded spherical SA to extend the sensing directions of damage monitoring in concrete structures. The feasibility of the SA sensors has been successfully validated for evaluating concrete strength, compressive stress, and internal damage [13,14]. Despite that, manual frequency point searches and unclear resonant frequency trends under damage effects remain challenging for those sensors. To address these issues, Pham et al. [15] proposed a new piezoelectric device known as a capsule-like smart aggregate (CSA), which can be easily designed to operate within any desired frequency range. Their preliminary study showed that variations in EMI signals of the CSA could directly reflect changes in concrete stress or the development of damage near the sensor’s region.

Despite its advantages, the primary challenges of the SA-based damage monitoring technique lie in handling the various phases of data gathering, processing the collected information, and precisely evaluating the presence and extent of structural damage. Until now, EMI features such as root-mean-square deviation (RMSD) and cross-correlation deviation (CCD) have been commonly used to estimate stress and detect damage status. However, these conventional multi-step processes require a rigorous feature extraction process to ensure accurate quantitative estimates of stress and damage [16,17,18,19]. A biased selection of EMI features, inadequate expert analysis, and erroneous decisions influenced by human intervention can lead to false damage alerts. For example, Nguyen et al. [17] quantify damage to a prestressed reinforced concrete girder by investigating the changes of statistical indices (i.e., RMSD and CCD) in each subrange of the raw EMI signal. The RMSD and CCD metrics showed distinguishable variations in some damage scenarios, while unexpected fluctuations were observed in others. In another study, Nguyen et al. [18] demonstrated that utilizing the statistical indices to quantify damage was challenging due to non-linear characteristics in the EMI signals under damage conditions. In addition, Zagrai et al. [19] investigated crack presence in thin aluminum plates using the EMI technique. The effect of opening the crack makes EMI spectra decrease but not uniform for all frequency bands. Those studies indicated that selecting sensitive frequency bands and meaningful EMI features is difficult and can impact the accuracy of evaluation results.

To address the above-mentioned issues, an automatic process for EMI feature extraction should be implemented for SA-based damage monitoring. Recently, the use of convolutional neural network (CNN)-based deep learning algorithms has proven effective in assessing the damage conditions of civil infrastructures [18,20,21]. Unlike machine learning methods, the architecture of the CNN algorithm is uniquely designed for feature extraction and damage detection in one procedure, which provides the capability to automatically process and learn the optimal features from raw data [21]. Some researchers have focused on combining CNN algorithms with the EMI technique for monitoring the health of concrete structures. Ai et al. [22] introduced a CNN-based deep learning model for concrete damage classification. The model was developed to identify compressive stress and load-induced cracking damage in concrete cubic structures. Their results demonstrated that the proposed CNN model achieved high accuracy, even when assessing minor damage. A few studies have used CNN-based regression methods for concrete damage monitoring. Li et al. [23] utilized a 2D CNN-based regression model learned EMI responses for quantitative monitoring of the concrete strength development in real time. Ta et al. [24] integrated the SA-based EMI technique and the 1D CNN model to quantitatively estimate stress in concrete structures. The estimated stresses in SA-embedded concrete cylinders could be automatically predicted with small errors, even for noise data. The aforementioned research has shown the effectiveness of using CNN-based deep learning techniques for monitoring stress and damage in concrete structures. 

Despite the previous research efforts, there exist two remaining issues related to the CSA-based EMI technique. Firstly, the peak frequency of the EMI signals rises with an increase in compressive forces, as reported in our previous work [15]. Linear relationships were observed between the statistical impedance features (i.e., RMSD and CCD indices) and the compressive stresses of the CSA. While the newly developed CSA [15] has demonstrated significant potential for generating highly sensitive EMI responses, preliminary validation has only been conducted on unconstrained CSAs. The performance and the sensitivity of EMI responses for the CSA embedded in a concrete medium, where they are fully constrained, have not been reported in the literature. Secondly, the implementation of a CNN-based regression algorithm to analyze the EMI response data of the newly developed CSA devices for predicting concrete stress has not yet been explored, with the new sensitivity of EMI signals measured by the constrained CSA sensors.

To address these research gaps, this study presents a method for monitoring stress variation using 1D CNN deep regression learning of EMI signals measured by a CSA sensor embedded in a concrete cylinder. First, the CSA-based EMI technique is described. A prototype of the CSA sensor is presented, and a theoretical EMI measurement model for the CSA sensor embedded in a concrete cylinder is described. The 1D CNN is developed to adapt and extract features of raw CSA’s EMI responses for stress estimation. Secondly, a uniaxial compression experiment on a CSA-embedded cylindrical concrete structure is conducted to record EMI responses under different stress levels. Finally, the performance of the 1D CNN model on stress regression is tested under the effects of noise-contaminated EMI and untrained stress EMI cases.

## 2. CSA-Based EMI Technique

### 2.1. Prototype of Capsule-like Smart Aggregate

**Figure 1:**
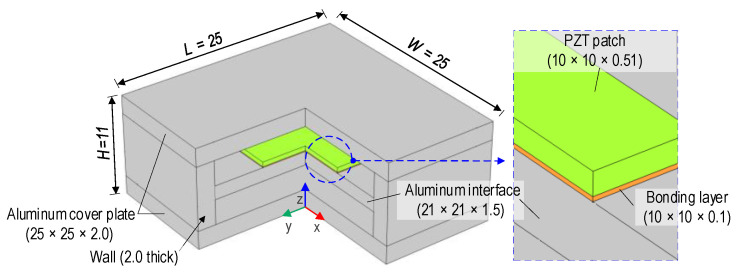
Prototype of CSA sensor (dimensions in mm).

A capsule-like smart aggregate (CSA) sensor [15] was developed based on the concept of the PZT interface technique [25], aiming to overcome the previous limitations of smart aggregate sensors [10,11,12]. The prototype of the CSA sensor (length (L) = 25 mm, width (W) = 25 mm, and height (H) = 11 mm) is shown in Figure 1. The CSA sensor consists of a PZT patch with length × weight × thickness of 10 × 10 × 0.51 mm, an aluminum interface plate with the size of 21 × 21 × 1.5 mm, a rectangular wall frame with thickness of 2 mm and height of 7mm, and two aluminum cover plates (i.e., top and bottom plates) the size of 25 × 25 × 2.0 mm.

The PZT material, PZT-5A, is selected based on its sensitivity in detecting damage to structures [26]. The aluminum interface plate is rigidly fixed to the wall frame. The geometrical and material properties of the interface plate can pre-determine the sensitive frequency range for the PZT sensor [27]. The PZT is surface-mounted in the middle of the interface with an epoxy glue layer, a so-called bonding layer, with a thickness of 0.1 mm. The top and bottom aluminum cover plates are glued to the interface to protect the PZT sensor from the concrete water in the CSA-embedded concrete fabrication process.

### 2.2. EMI Measurement Model for CSA–Concrete Interactive System

**Figure 2:**
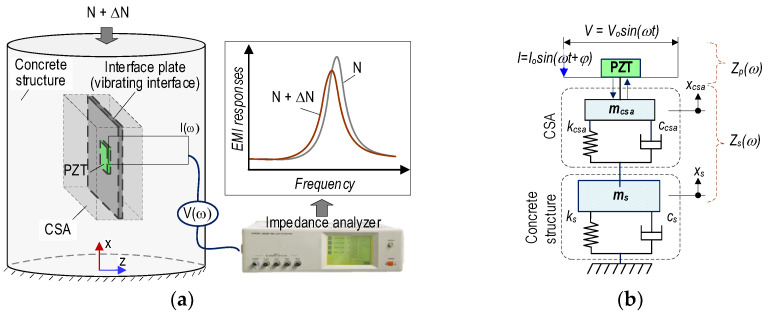
CSA-based EMI measurement 2 DOFs model for concrete structure: (**a**) CSA-embedded concrete structure; (**b**) 2 DOFs model.

Figure 2a illustrates a CSA-based EMI measurement model for the cylindrical concrete structure under compressive load N. The CSA sensor embedded in a concrete structure is activated via an electrical voltage *V*(*ω*) with current *I*(*ω*). The excited voltage generates a mechanical strain on CSA and CSA’s closed region via the inverse piezoelectric effect. At the same time, the electrical signal from structure responses is also recorded by the PZT patch via a direct piezoelectric effect. Thus, under variation of compressive load (i.e., ΔN), any changes in concrete structure could lead to changes in EMI signature.

Figure 2b shows a 2 degrees of freedom (2 DOFs) EMI model between the coupling motions of the CSA sensor and the monitored structure, following the reference [28]. The coupled structural mechanical impedance *Z_S_* of the CSA and the host structure can be presented as follows:

(1) $$Z_{s}\left(\omega \right)=\frac{T_{11}\left(\omega \right)T_{22}\left(\omega \right)-T_{12}^{2}(\omega )}{i\omega T_{22}\left(\omega \right)}$$

where the dynamic stiffness parts, T*_xy_* (*x*, *y* = 1–2), depend on the structural features of the CSA and the investigated host structure.

Equation (2) shows a function, *Z*(*ω*), of the PZT sensor’s structural mechanical impedance and CSA-host structure.

(2) $$Z\left(\omega \right)={\left\{i\omega \frac{w_{p}l_{p}}{t_{p}}\left({\hat{\varepsilon }}_{33}^{T}-\frac{1}{\frac{Z_{p}\left(\omega \right)}{Z_{s}\left(\omega \right)}+1}{d_{31}^{2}\hat{Y}}_{11}^{E}\right)\right\}}^{-1}$$

where the width, thickness, and length of the PZT patch are $w_{p}$, $t_{p},$ and $l_{p}$ orderly; ${\hat{\epsilon }}_{33}^{T}$ is the complex dielectric constant; $Z_{p}\left(\omega \right)={\hat{Y}}_{11}^{E}w_{p}t_{p}/(j\omega l_{p})$ is the structural mechanical impedance of the PZT patch;$\;d_{31}$ is the constant of the PZT sensor at zero stress; ${\hat{Y}}_{11}^{E}$ signifies the intricate Young modulus of the PZT plate under the zero electric field condition. As depicted in Equation (2), the PZT’s structural mechanical impedance, $Z_{p}\left(\omega \right)$, and the CSA-host structure, $Z_{s}\left(\omega \right)$, were involved in the real part of impedance, $Z\left(\omega \right)$. Hence, variations in structural properties due to stress or damage could affect the EMI responses. 

**Figure 3:**
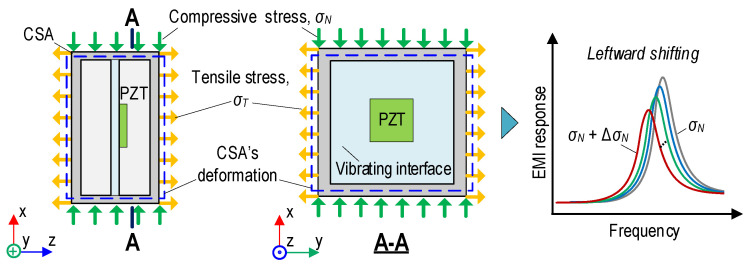
Behavior of CSA sensor in x-direction embedded in concrete cylinder under compression.

The behavior of a CSA sensor embedded in the concrete structure is illustrated in Figure 3. Under the x-directional compressive stress $\sigma_{N}$, the sensor experiences stress–strain responses. The vibrating interface of the CSA sensor experiences compressive deformation in the x-direction. 

Due to Poisson’s effect, the other surfaces of the sensor, such as z- and y-directions, are subjected to expansion under tensile stress $\sigma_{T}$. It is observed that the expansion of the vibrating interface induced by the tensile stress would change the structural impedance. Therefore, increasing the applied stress on the CSA-embedded concrete structure could result in a leftward shift in the EMI responses; see Figure 3.

## 3. Design of 1D CNN Deep Learning Method for CSA-Based Concrete Stress Evaluation

### 3.1. 1D CNN-Based Concrete Stress Monitoring Method

**Figure 4:**
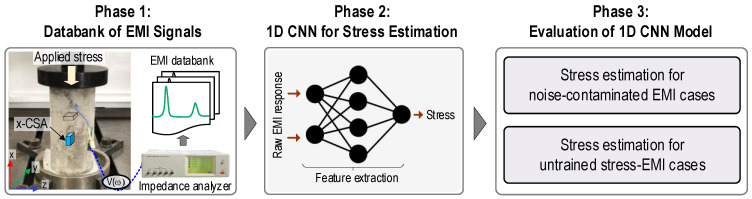
Diagram of 1D CNN stress estimation model using CSA’s EMI signals.

Figure 4 presents the overall scheme for concrete stress monitoring via 1D CNN deep regression learning of CSA’s EMI data. It includes three phases: (1) configuration of CSA’s EMI databank, (2) development of 1D CNN model for stress estimation, and (3) evaluation of 1D CNN model. In Phase 1, a series of raw EMI signals and their related structural attributes (e.g., stress levels and severity of damage) are collected to construct datasets for monitoring stress in a CSA-embedded concrete cylinder. In Phase 2, the 1D CNN model is developed to adaptively process CSA’s EMI signals for concrete stress estimation. In Phase 3, the 1D CNN model is evaluated for noise-contaminated EMI cases and untrained stress EMI cases.

**Figure 5:**
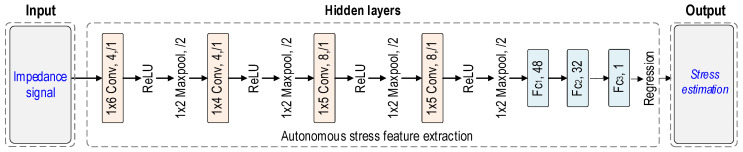
Architecture of 1D CNN stress estimation model using EMI signals [24].

The 1D CNN architecture from our previous study [24] was utilized to process CSA’s EMI responses for stress estimation. As shown in Figure 5, three parts of a 1D CNN model include input, hidden layers, and output. To process the CSA sensor’s EMI signals, the 1D CNN architecture modified the input to receive N × 225 input data, where N represents the EMI data number. There are 225 measurement points within the frequency bands of each signal (see Section 4.2). The hidden layers are structured with convolutional (Conv) layers with weight kernels, rectified linear unit (ReLU) layers, max pooling (Maxpool) layers, fully connected (Fc) layers, and a regression output layer. The output layer outputs the estimated stress value in the MPa unit. The total training parameters of the 1D CNN model are 6081 parameters. Table 1 shows the specifications of the 1D CNN layers.

### 3.2. Databank of EMI Signals for CNN Model Evaluation

Variations in EMI signals can occur due to factors such as sensor configuration and sensor bonding [29]. The execution of comprehensive experiments encompassing all these variables poses challenges and incurs costs. Therefore, data augmentation by injecting Gaussian noise into the measured EMI signals is a practical alternative for simulating realistic measurement conditions. 

The formula for Gaussian noise simulation into EMI signals is shown in Equation (3). The term *x*[*n*] is the EMI signal contaminated with noise; *s*[*n*] denotes the measured amplitude of the EMI; *w*[*n*] stands for Gaussian noise; and *n* is the number of investigated measurement points.

(3) $$x\left[n\right]=s\left[n\right]+w[n]\times s[n]$$

Note that Gaussian noise simulation based on the mentioned formula cannot be used to consider EMI signals’ variability induced by the temperature. In fact, the behavior of EMI signals via a PZT interface (i.e., CSA’s vibrating plate) is very different. As shown in our previous work [30], the results of quantification of the temperature effect caused the shifting in the EMI signal’s frequency and amplitude. Meanwhile, the effect of Gaussian noise only caused noises in the EMI’s amplitude (see Equation (3)).

**Figure 6:**
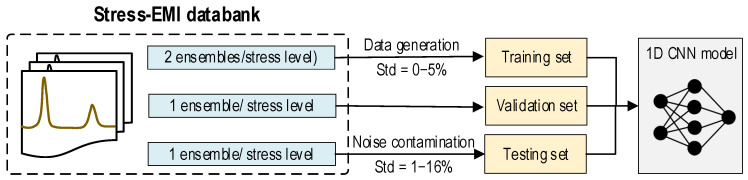
Data configuration for noise-contaminated EMI cases.

Data configuration for noise-contaminated EMI cases is shown in Figure 6. The performance of the 1D CNN model is evaluated by using data sets (i.e., training, validation, and testing sets) created from the recorded stress EMI databank. The EMI response is recorded in four ensembles for each compressive stress level. Gaussian noise with standard deviations of 0%, 1%, 2%, 3%, 4%, and 5% of the signal amplitude is added to the first two ensembles to create the training set. The third ensemble is used to monitor overfitting [31] during the training of the model. The testing set is constructed by injecting noise levels ranging from 1% to 16% (with a 1% increment) into the fourth ensemble of each stress level. The effect of noise-contaminated EMI cases on the accuracy of the 1D CNN model is extensively examined for a concrete cylinder, as presented in Section 5.1.

When trained on limited data, convolutional neural network-based deep learning techniques regularly struggle with stability and accuracy [31]. A well-designed deep learning model is one that can perform well even with small datasets [32]. To assess this, we reduced the training data to evaluate the performance of the 1D CNN model in predicting untrained stress-EMI cases.

**Figure 7:**
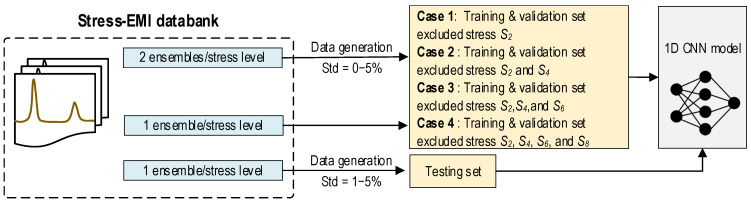
Data configuration for untrained stress-EMI cases.

As shown in Figure 7, we added noise (0% to 5% of signal magnitude) to the first two ensembles to create the training data sets. The third ensemble at each stress level was utilized for validation. To form the testing data sets, the fourth ensemble was injected with noises from 1% to 5% with an interval of 1%. We created two testing scenarios to examine the effect of untrained data on the 1D CNN’s performance. In case 1, the training and validation sets were excluded from stress level S_2_ (from eight levels S_1_–S_8_). In case 2, the training and validation sets were excluded from stress levels S_2_ and S_4_. In case 3, the training and validation sets were excluded from stress levels S_2_, S_4_, and S_6_. In case 4, the training and validation sets were excluded from stress levels S_2_, S_4_, S_6_, and S_8_. The effect of untrained stress EMI cases on the accuracy of the 1D CNN model was extensively examined for a concrete cylinder, as presented in Section 5.2.

## 4. Experimental Test

### 4.1. Fabrication of CSA-Embedded Concrete Cylinder

**Figure 8:**
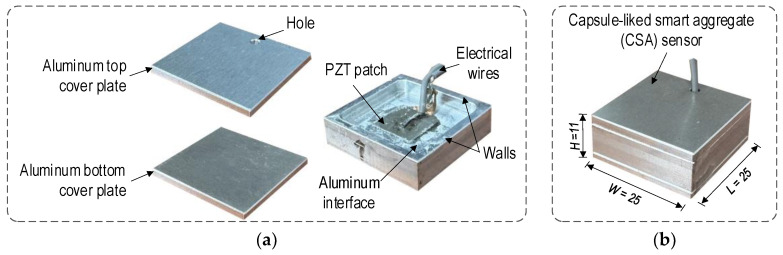
CSA prototype (dimensions in mm): (**a**) CSA sensor’s components; (**b**) Fabricated CSA.

Figure 8 shows a prototype of the CSA sensor. The CSA sensor was constructed by assembling an aluminum interface plate (21 × 21 × 1.5 mm) rigidly fixed to wall frame and two aluminum cover plates (25 × 25 × 2 mm). In the interface, a PZT patch (10 × 10 × 0.51 mm) was adhered to the center. The top cover plate had a drilled hole with a diameter of 2 mm to allow electric wires to pass through. Figure 8b shows the complete version of the CSA sensor for embedding in concrete structures.

**Figure 9:**
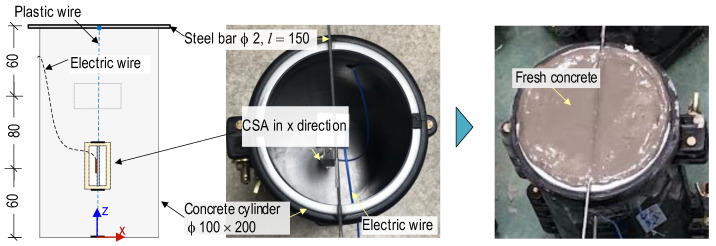
Fabrication of CSA-embedded concrete cylinder (dimensions in mm).

Figure 9 shows the fabrication process of a CSA-embedded concrete cylinder. The CSA was positioned in a standard plastic cylinder the size of 100 × 200 mm. An x-CSA sensor was oriented with the planar surface of the PZT parallel to the applied force under compression. The x-CSA sensor was positioned 60 mm from the bottom. A plastic wire (length 150 mm) and a steel bar (ϕ2 mm) were used to fix the sensor to the cylinder mold. The concrete components were weighted using a digital scale. Then, they were evenly mixed by hand and poured into the cylinder molds. After 48 h of concrete casting, the molds were removed, and the concrete samples were cured with wet blankets for 28 days under normal conditions. The age of the concrete samples under compression test was 28 days. Table 2 describes the material properties of the CSA-embedded concrete cylinder.

### 4.2. Experimental Setup

**Figure 10:**
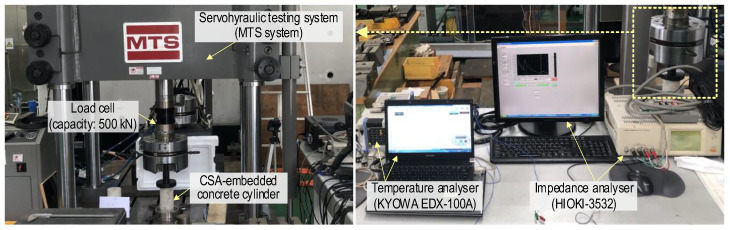
Testing setup for EMI measuring from x-CSA-embedded concrete cylinder under compression.

Figure 10 shows the test setup to measure the EMI responses of the CSA-embedded concrete cylinder. As shown in the figure, a concrete sample was placed inside a load frame of a servo-hydraulic materials test system (MTS system). A load cell with a 500 kN capacity was utilized to monitor the actual compression force. EMI signals from the CSA sensor were recorded using a HIOKI 3532 impedance analyzer. Room temperature was also tracked during the experiment with a KYOWA EDX-100A.

**Figure 11:**
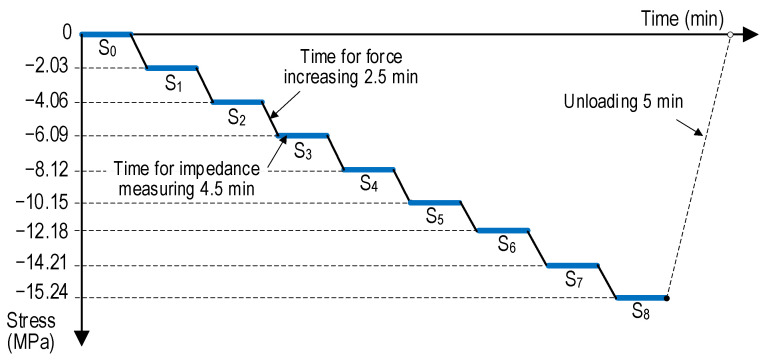
Applied loading history on x-CSA-embedded cylinder.

Figure 11 depicts nine loading scenarios for the cylinder, from S_0_ = 0 MPa to S_8_ = 15.24 MPa. Specifically, the applied stress levels from S_0_ (0 MPa) to S_7_ (14.21 MPa) increased with a value of 2.03 MPa (i.e., an increasing rate of 14.28%). The stress level S_8_ was 15.24 MPa, about 60% of compressive strength σ_c_ (see Table 2). The nine loading scenarios were set to investigate the behavior of the embedded CSA sensor in the linear working domain of concrete material. The stress levels were controlled by MTS multipurpose test software version 793. The time for force increasing was 2.5 min. The time for impedance measuring was 4.5 min. The loading rate was a constant speed of 0.0113 MPa/s^−1^. The EMI signals were measured with 225 points in the 15–26 kHz range. The monitored temperature in this experiment varied between 22 °C and 23 °C.

**Figure 12:**
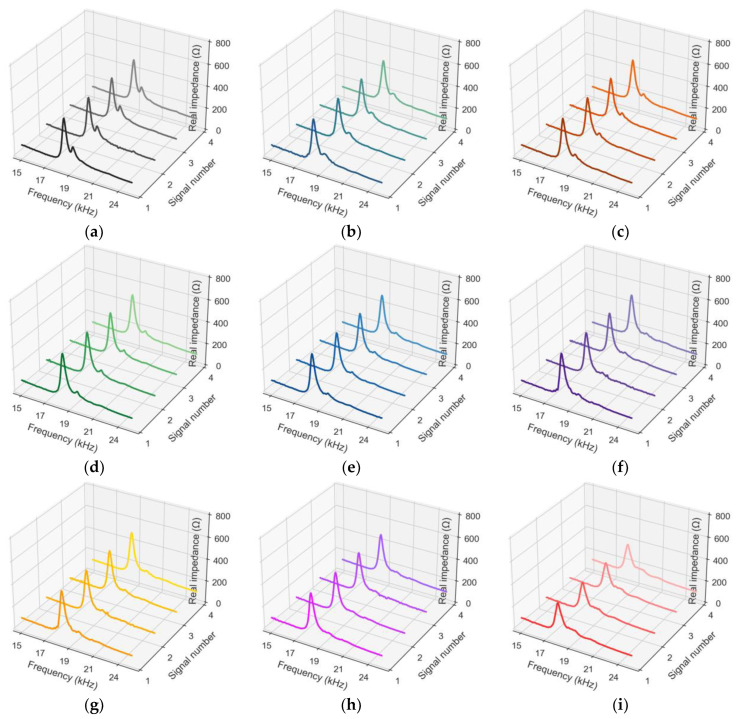
EMI responses (in average of ensembles) of CSA in cylinder under applied stresses S_0–8_: (**a**) S_0_; (**b**) S_1_; (**c**) S_2_; (**d**) S_3_; (**e**) S_4_; (**f**) S_5_; (**g**) S_6_; (**h**) S_7_; (**i**) S_8_.

Figure 12 shows measured EMI responses under applied stresses S_0–8_. In each stress level, four EMI measurements (i.e., four ensembles) were recorded for a frequency range from 15 kHz to 26 kHz. In Figure 12, a total of 36 EMI responses were recorded under eight stress levels S_0_–S_8_. In Figure 12a, four ensembles at applied stress S_0_ are plotted. These signals were captured for the frequency range 15–26 kHz. It was noted that, at the range of 15–26 kHz, the EMI responses (i.e., EMI spectrum) of the CSA sensor in the cylinder had a prominent peak with the highest magnitude, representing meaningful structural information [15]. Thus, the 15–26 kHz frequency range was selected to experimentally investigate the sensitivity of embedded CSA’s EMI responses to compression loading. 

**Figure 13:**
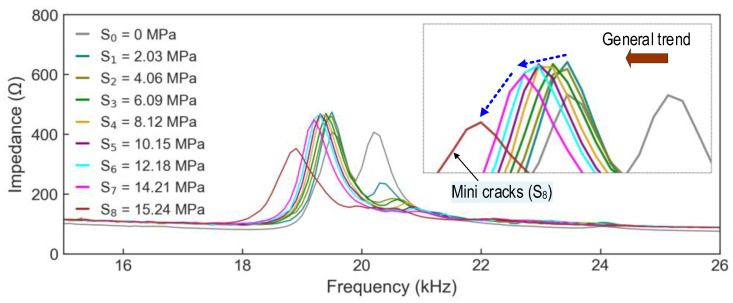
EMI responses (in average of ensembles) of CSA in cylinder under applied stresses S_0_–S_8_.

Under levels of applied stress (see Figure 12b–i), it was observed that the resonant peak in the frequency domain varied significantly along with real impedance magnitude. As shown in Figure 13, an EMI plot (in average of ensembles) was provided for a deeper evaluation relevant to the trends of the resonant frequency shift and real impedance magnitude under the applied stress levels. Compared with the previous study [15], it was found that the peak amplitude of the CSA embedded in the concrete medium was significantly lower than that of the unembedded CSA sensor. This amplitude difference reflects the effect of increasing damping induced by the concrete medium. Also, the peak frequency of the embedded CSA in this study was lower than that of the unembedded CSA obtained from the previous study. The frequency reduction may have been caused by the effect of added mass when the CSA was constrained by the surrounding concrete. Under compressive loading conditions, the EMI spectra for both embedded and unembedded CSA sensors experienced leftward shifts. This shifting trend was consistent with the theoretical explanation in Figure 3.

**Figure 14:**
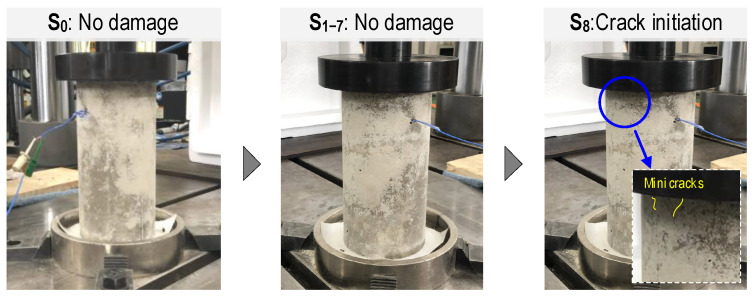
Visual observation of cylinder during loading steps S_0_–S_8_.

**Figure 15:**
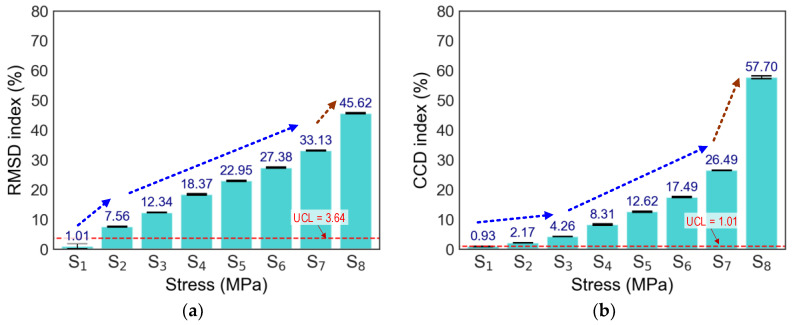
EMI features of x-CSA under applied stresses: (**a**) RMSE; (**b**) CCD.

From the observation of the damage status of the cylinder, small cracks were found at loading case S_8_, as visualized in Figure 14. Figure 15 presents the computed statistical features (i.e., RMSD and CCD indices) to quantify variations in raw EMI signals under stress levels S_1_–S_8_. To support decision-making, the upper control limit (UCL) was calculated as three standard deviations from the mean (99% confidence level), so any indices exceeding the UCL indicated changes in stress levels. Figure 15a shows the RMSD indices of the CSA corresponding to all stress levels applied to the cylinder. The RMSD indices linearly increased from the stress level S_1_ to S_7_, and the RMSD indices changed abruptly under S_8_. Figure 15b shows the CCD indices of the CSA sensor. The magnitudes of CCD increased non-linearly under increasing applied stress levels (S_1_–S_7_). The magnitudes of CCD non-linearly increased for the applied stress levels (S_1_–S_7_). It was observed that both RMSD and CCD indices for the embedded CSA case increased with the increase in the applied stress levels. 

In the previous work [15], the CSA sensor itself (which was not embedded in concrete) was compressed at different loadings. The CSA’s vibrating plate was positioned parallel to the x-directional loadings in the compression experiment. The CSA’s vibrating plate was rigidly fixed on two opposite sides (i.e., two sides could not move or rotate), and the other two sides were free to move and deform. In contrast with the unembedded CSA case [15], the correlation between these EMI features and the concrete stress was non-linear. The CCD metric exhibited more non-linear changes with the concrete stress than the RMSD metric. The CCD metric showed a sudden increase at the stress level S_8_, indicating that the concrete was damaged with some micro cracks, as observed in Figure 14.

## 5. Stress Evaluation of 1D CNN Model

### 5.1. Stress Estimation for Noise-Contaminated EMI Cases

#### 5.1.1. Data Preparation

The stress EMI data obtained from compressive tests on the x-CSA-embedded concrete cylinder was used to create the databank for the 1D CNN model. Eight labels of “stress” were labeled to the EMI data, as shown in Table 3. For the first seven stress levels (from S_1_ to S_7_), the stress values starting from 2.03 MPa to 14.21 MPa with an increasing step of 2.03 MPa were labeled to the EMI data, respectively. The label for the eighth level (S_8_) was labeled as 15.24 MPa.

The training data sets were created by injecting Gaussian noise (with standard deviations ranging from 0% to 5% of the signal amplitude) into the first two ensembles, producing 384 signals for 8 stress levels. To form the validation set, the third ensemble was employed, and the set had 8 signals. To form the testing dataset, noise levels from 1% to 16% were added to the remaining raw signal at each stress level. For each noise level, 10 new signals were generated for each of the eight stress levels, resulting in a total of 1280 signals for the 16 noise levels. Consequently, the testing dataset comprised 1288 signals, including the original 8 raw EMI signals.

**Figure 16:**
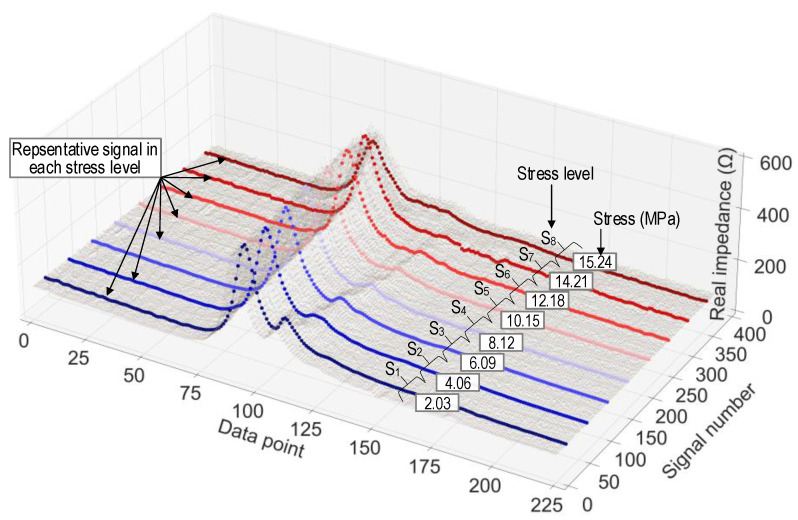
Visualization of labeled EMI data in training set.

**Figure 17:**
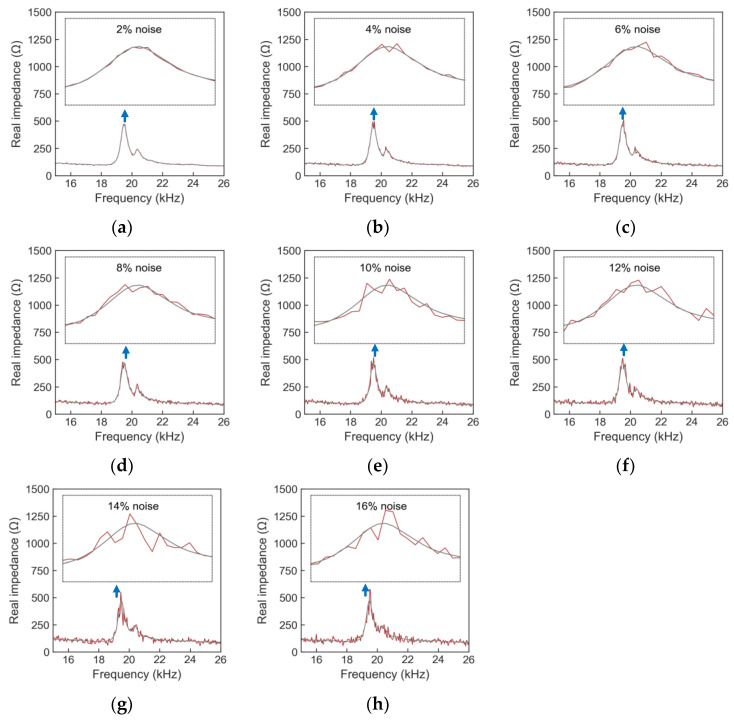
Example of noise-contaminated EMI signals under stress level S_1_ in testing set: (**a**) 2%; (**b**) 4%; (**c**) 6%; (**d**) 8%; (**e**) 10%; (**f**) 12%; (**g**) 14%; (**h**) 16%.

Figure 16 illustrates the labeled EMI signals in the training set. Each signal in the figure consists of 225 data points. With each stress level, 10,800 data points were recorded; thus, for 8 stress levels, a total of 86,400 data points were acquired. Figure 17 shows an example of EMI signals contaminated with noise at stress level S_1_ within the testing set. A total of 16 levels of noise (1~16%) were added to the raw signals.

#### 5.1.2. Training Results

A desktop computer with a GeForce GT 2080 Ti GPU (11 GB), Intel Core i9-9000KF CPU (3.6 GHz), and 64 GB of RAM was used for all the computations of deep learning works. The 1D CNN models were built using Python and trained using the Adam optimizer with a mini-batch size of 2 and a learning rate of 0.0001.

**Figure 18:**
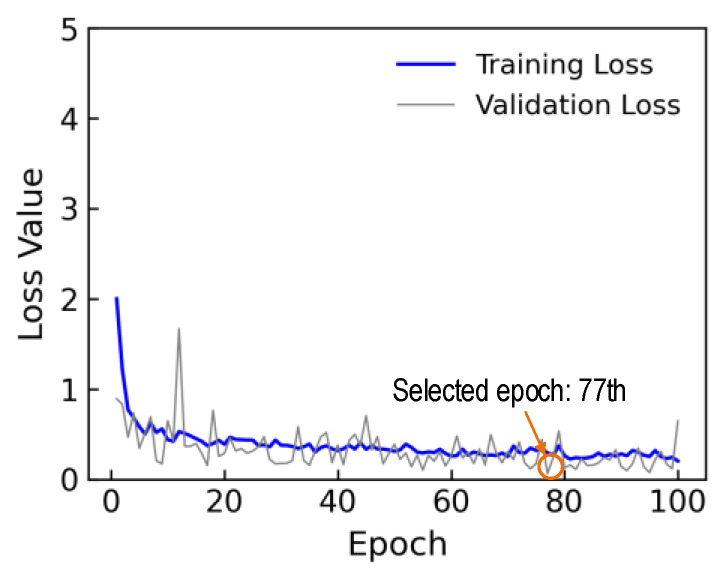
Loss values after 100 epochs.

Equation (4) presents the loss function of the regression layer, where N indicates the total number of data. Also $\sigma_{i}$ and ${\hat{\sigma }}_{i}$ represent the estimated stress and the actual stress for the ith EMI data. The loss values of the 1D CNN model after completing a 100-epoch training procedure are plotted in Figure 18. The training loss significantly decreased within the first six epochs, followed by a gradual convergence over subsequent epochs. The validation loss experienced fluctuation without convergence until the 100th epoch. The training time for 6081 parameters of 1D CNN was about 64.5 s. The trained 1D CNN model at the 77th epoch, corresponding to the minimum value of the loss validation in the 100 epochs, was chosen to evaluate its accuracy on the testing set.

(4) $$Loss=\frac{1}{N}\sum_{i=1}^{N}\left|\sigma_{i}-{\hat{\sigma }}_{i}\right|$$

#### 5.1.3. Testing Results

**Figure 19:**
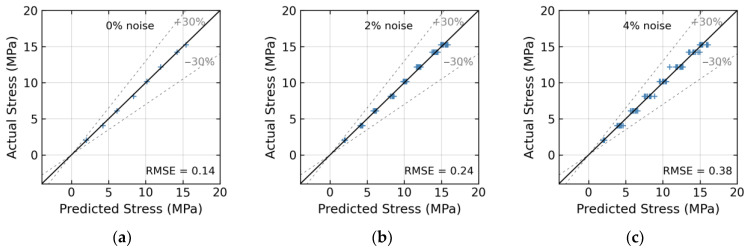

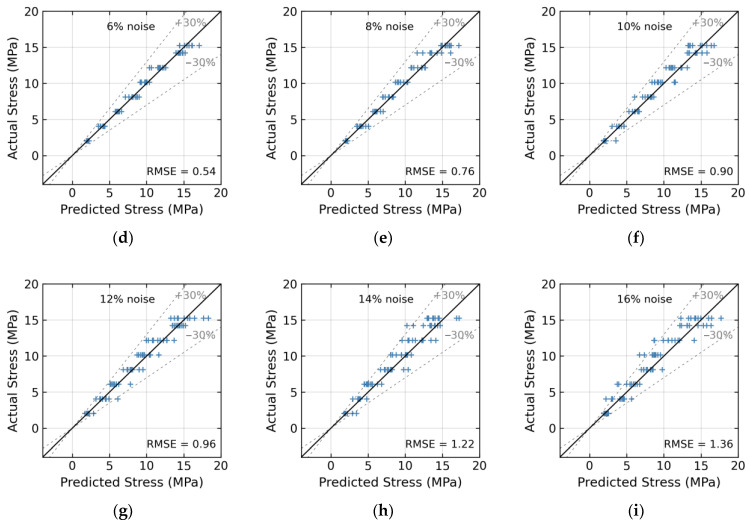
(**a**) 0%; (**b**) 2%; (**c**) 4%; (**d**) 6%; (**e**) 8%; (**f**) 10%; (**g**) 12%; (**h**) 14%; (**i**) 16%.

As shown in Figure 19, the relationship between the estimated and actual stress values is plotted for various noise levels. Equation (5) shows the root mean square error (RMSE) index which was utilized to evaluate the accuracy of the trained 1D CNN regression model on the testing dataset. The RMSE measured the average of the squares of the errors between estimated stress and actual stress. The RMSE error indicated the stress estimation errors in terms of stress monitoring, with a unit in MPa.

(5) $$RMSE=\sqrt{\frac{1}{N}\sum_{i=1}^{N}{\left(\sigma_{i}-{\hat{\sigma }}_{i}\right)}^{2}}$$

In Figure 19a, the model precisely predicted stress values with an RMSE error of about 0.14 at 0% noise level. In Figure 19d, the model precisely predicted stress values with the RMSE error of about 0.54 at a 6% noise level. At a noise level greater than 10%, the estimated stress error of 1D CNN was over 30%.

**Figure 20:**
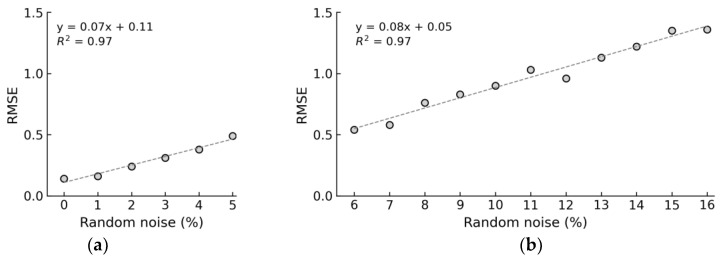
RMSE error under noise levels: (**a**) levels of noise 0–5% (trained levels); (**b**) levels of noise 6–16% (untrained levels).

Figure 20 presents relationships between the RMSE and noise levels. The analyzed results are divided into two phases. The first phase includes levels of noise from 0% to 5%, representing the phase of trained levels. The second phase includes levels of noise from 6% to 16%, representing the phase of untrained levels. In Figure 20a, the RMSE was 0.14 at 0% noise level and 0.49 at 5% noise level. In Figure 20b, the RMSE was 0.54 at a 6.0% noise level and 1.36 at a 16% noise level. At noise levels from 0% to 5%, the RMSE values rose almost linearly. At noise levels from 6% to 16%, the RMSE values rose with fluctuations (i.e., rose non-linearly). It is shown that the increase of prediction error in the case of untrained noise levels was notably greater than that of trained noise levels, as demonstrated by the gradients of the linear approximation functions in Figure 20a,b. Overall, the stress estimation of the 1D CNN model became less accurate as the noise level increased.

### 5.2. Stress Estimation for Untrained Stress EMI Cases

#### 5.2.1. Data Preparation

The untrained stress EMI case was established based on the data configuration scheme shown in Figure 7. A total of 32 signals were obtained from stress levels from S_1_ to S_8_. For the training set, the first two ensembles from each stress level were used, yielding a total of 384 signals across the 8 stress levels. For the validation set, the third ensemble from each stress level was utilized. A total of 8 signals were involved in the validation set. The final ensemble was used to create the testing set, comprising 400 signals with noise ranging from 1% to 5% and 8 signals with no noise.

**Figure 21:**
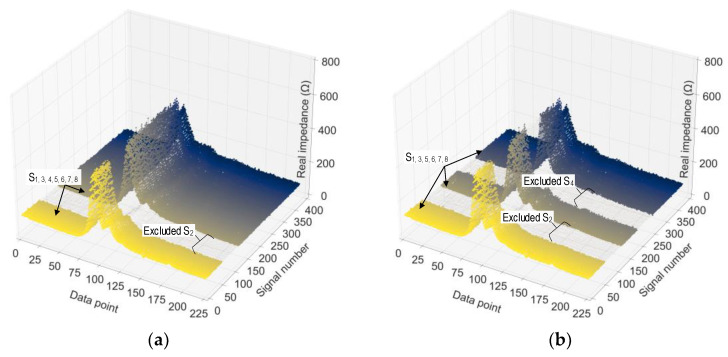
Training data in untrained cases: (**a**) untrained case 1 (excluded stress S_2_); (**b**) untrained case 2 (excluded stress S_2_ and S_4_).

Four scenarios were established to investigate the effect of untrained stress EMI cases on the 1D CNN’s performance. Table 4 summarizes data sets for untrained stress EMI cases. In untrained scenario 1 (see Figure 21a), the stress level S_2_ (among eight levels S_1_–S_8_) was excluded from the training and validation sets. After omitting 48 signals from the training set and 1 from the validation set, the remaining signals were 336 in the training set and 7 in the validation set. In untrained scenario 2, the stress levels S_2_ and S_4_ were excluded from the training and validation sets. After omitting 96 signals from the training set and 2 from the validation set, the remaining signals were 288 in the training set and 6 in the validation set. In untrained scenario 3, the stress levels S_2_, S_4_, and S_6_ were excluded from the training and validation sets. After omitting 144 signals from the training set and 3 from the validation set, the remaining signals were 240 in the training set and 5 in the validation set. In untrained scenario 4, the stress levels S_2_, S_4_, S_6_, and S_8_ were excluded from the training and validation sets. After omitting 192 signals from the training set and 4 from the validation set, the remaining signals were 192 in the training set and 4 in the validation set.

**Figure 22:**
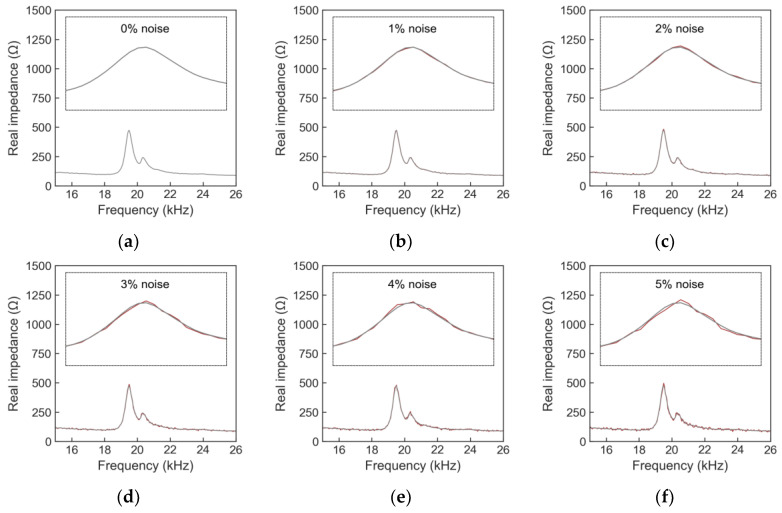
Example of noise-added EMI signals under stress level S_1_ in testing set: (**a**) 0%; (**b**) 1%; (**c**) 2%; (**d**) 3%; (**e**) 4%; (**f**) 5%.

The labeled EMI data in the training set in untrained cases are illustrated in Figure 21. In Figure 21a, untrained scenario 1, the stress level S_2_ was omitted from the training set. In Figure 21b, untrained scenario 2, the stress levels removed from the training data were S_2_ and S_4_. Figure 22 illustrates the noises in EMI signals at stress level S_1_ in the testing set.

#### 5.2.2. Training Results

**Figure 23:**
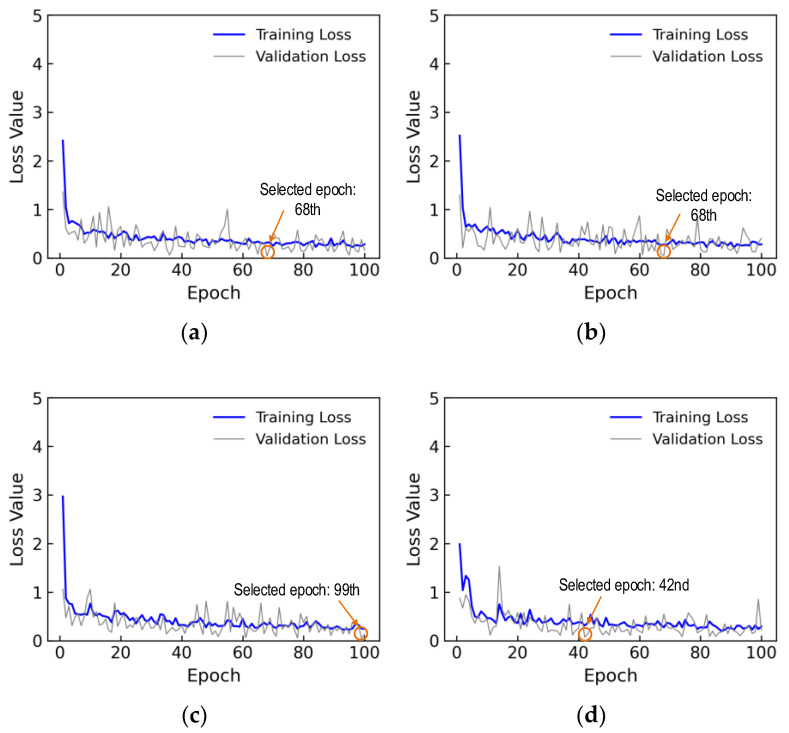
Loss values for untrained cases: (**a**) case 1 (excluded stress S_2_); (**b**) case 2 (excluded stress S_2_ and S_4_); (**c**) case 3 (excluded stress S_2_, S_4_, and S_6_); (**d**) case 4 (excluded stress S_2_, S_4_, S_6_, and S_8_).

Figure 23a plots the loss values of the 1D CNN model in a training process of 100 epochs for the untrained case 1. In the figure, the training loss significantly reduced in the first 4 epochs, then gradually decreased with a few small ups and downs until the 60th epoch. The loss remained stable until the 100th epoch. For the first 18 epochs, the validation loss declined with high variations. Then, the validation loss fluctuated slightly until the learning process ended. It was observed that the training loss value was approximately 0.32 at the 68th epoch, corresponding to the lowest validation loss of nearly 0.04. Thus, the trained 1D CNN model at the 68th epoch was chosen to investigate its accuracy on the testing set.

The loss values of the 1D CNN model during 100 epochs for the untrained scenario 2 are shown in Figure 23b. In the figure, the training loss rapidly drops in the first 4 epochs. The loss was reduced gradually with slight fluctuations until the 70th epoch, and then it remained stable until the 100th epoch. The validation loss experienced high fluctuations in the first 60 epochs. Afterward, the validation loss fluctuated slightly until the 100th epoch. The minimum value of the validation loss was 0.05 at the 68th epoch corresponding tothe training loss value of about 0.27. Thus, the trained 1D CNN model at the 68th epoch was chosen to investigate its accuracy on the testing set of the untrained scenario 2.

The loss values of the 1D CNN model during 100 epochs for the untrained scenario 3 are shown in Figure 23c. In the figure, the training loss rapidly drops in the first 5 epochs. The loss was reduced gradually with slight fluctuations until the 65th epoch, and then it remained stable until the 100th epoch. The validation loss experienced high fluctuations in the first 80 epochs. Afterward, the validation loss fluctuated slightly until the 100th epoch. The minimum value of the validation loss was 0.04 at the 99th epoch corresponding to the training loss value of about 0.24. Thus, the trained 1D CNN model at the 99th epoch was chosen to investigate its accuracy on the testing set of the untrained scenario 3.

The loss values of the 1D CNN model during 100 epochs for the untrained scenario 4 are shown in Figure 23d. In the figure, the training loss rapidly drops in the first 7 epochs. The loss was reduced gradually with slight fluctuations until the 80th epoch, and then it remained stable until the 100th epoch. The validation loss experienced fluctuations during 100 epochs. The minimum value of the validation loss was 0.08 at the 42nd epoch corresponding to the training loss value of about 0.31. Thus, the trained 1D CNN model at the 42nd epoch was chosen to investigate its accuracy on the testing set of the untrained scenario 4.

#### 5.2.3. Testing Results

**Figure 24:**
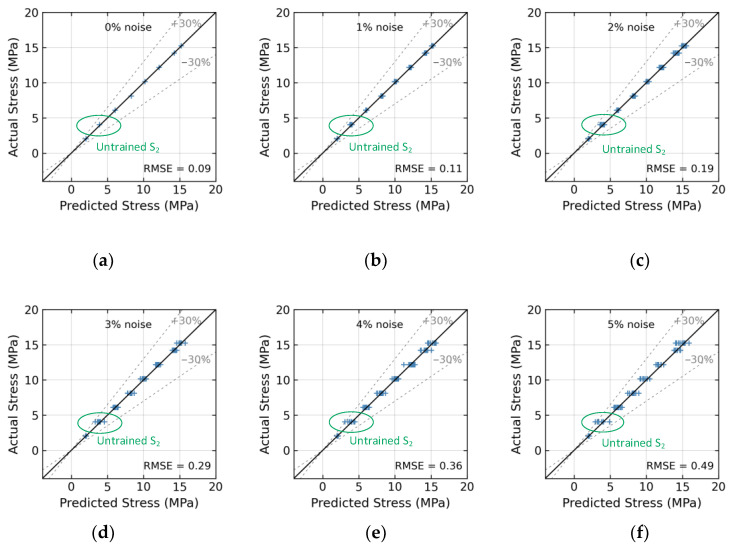
Stress estimation for untrained case 1 (excluded stress S_2_): (**a**) 0% noise; (**b**) 1% noise; (**c**) 2% noise; (**d**) 3% noise; (**e**) 4% noise; (**f**) 5% noise.

**Figure 25:**
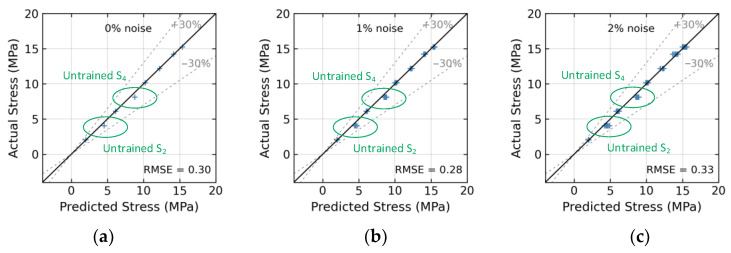

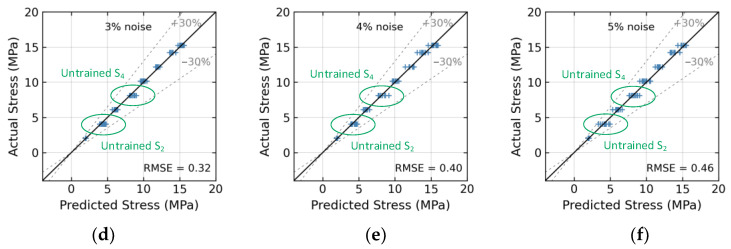
Stress estimation for untrained case 2 (excluded stress S_2_ and S_4_): (**a**) 0% noise; (**b**) 1% noise; (**c**) 2% noise; (**d**) 3% noise; (**e**) 4% noise; (**f**) 5% noise.

The effect of untrained stress S_2_ on the accuracy of the 1D CNN model is presented in Figure 24. At a 0% noise level (see Figure 24a), the model accurately predicted stress with an RMSE error of 0.09. In Figure 24d, with a 3% noise level, the model accurately predicted stress with the RMSE error of approximately 0.29. Figure 25 presents the effect of untrained stress levels S_2_ and S_4_ on the 1D CNN model’s accuracy. At a 0% noise level (see Figure 25a, with no noise), the model’s stress estimation achieved an RMSE error of 0.3. At a 4% noise level (see Figure 25e), the model’s RMSE error rose to approximately 0.4.

**Figure 26:**
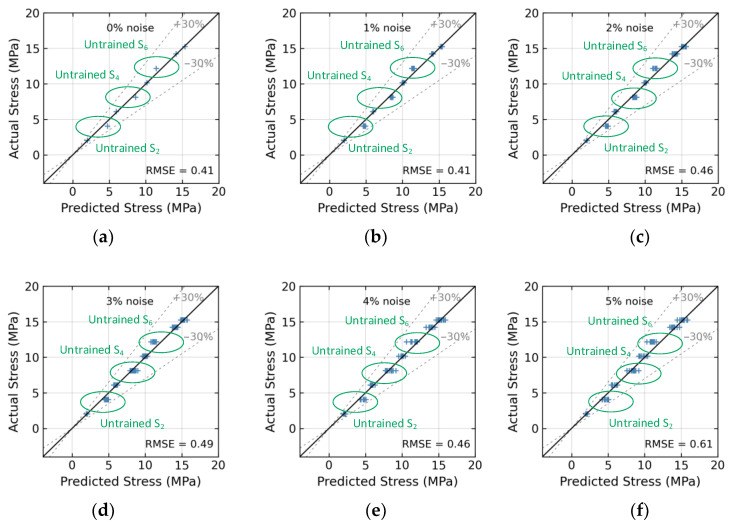
Stress estimation for untrained case 3 (excluded stress S_2_, S_4_, and S_6_): (**a**) 0% noise; (**b**) 1% noise; (**c**) 2% noise; (**d**) 3% noise; (**e**) 4% noise; (**f**) 5% noise.

**Figure 27:**
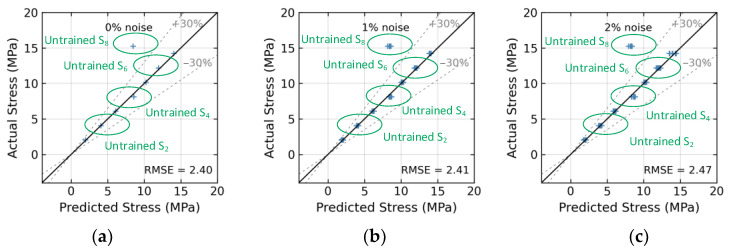

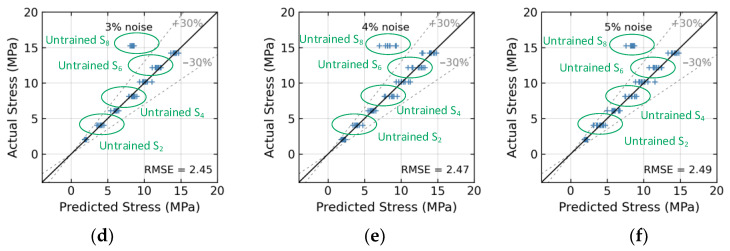
Stress estimation for untrained case 4 (excluded stress S_2_, S_4_, S_6_, and S_8_): (**a**) 0% noise; (**b**) 1% noise; (**c**) 2% noise; (**d**) 3% noise; (**e**) 4% noise; (**f**) 5% noise.

Figure 26 presents the effect of untrained stress levels S_2_, S_4_, and S_6_ on the 1D CNN model’s accuracy. At a 0% noise level (see Figure 26a, with no noise), the model’s stress estimation achieved an RMSE error of 0.41. At a 4% noise level (see Figure 26e), the model’s RMSE error rose to approximately 0.46. Figure 27 presents the effect of untrained stress levels S_2_, S_4_, S_6_, and S_8_ on the 1D CNN model’s accuracy. At a 0% noise level (see Figure 27a, with no noise), the model’s stress estimation achieved an RMSE error of 2.4. At a 4% noise level (see Figure 27e), the model’s RMSE error rose to approximately 2.47.

**Figure 28:**
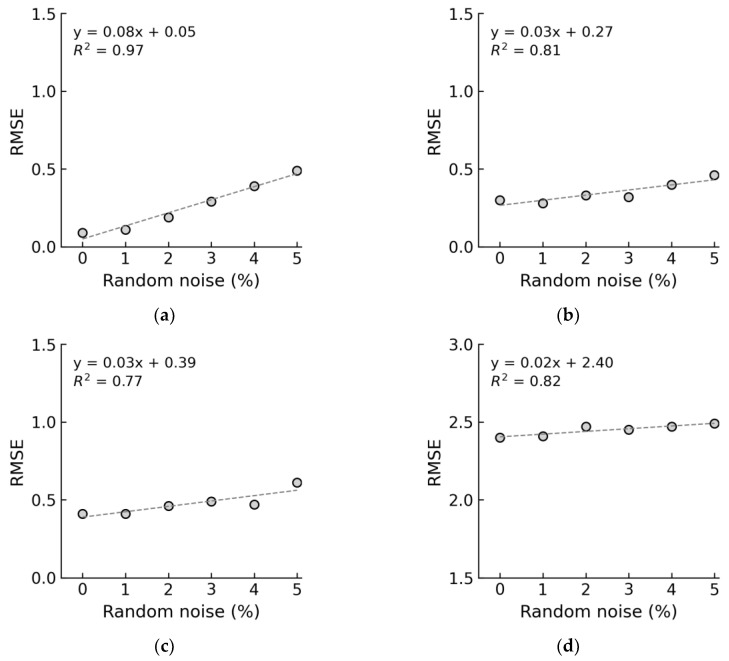
RMSE error in untrained cases: (**a**) untrained case 1 (excluded stress S_2_); (**b**) untrained case 2 (excluded stress S_2_ and S_4_); (**c**) untrained case 3 (excluded stress S_2_, S_4_, and S_6_); (**d**) untrained case 4 (excluded stress S_2_, S_4_, S_6_, and S_8_).

The relationships between RMSE and noise levels are shown in Figure 28. In Figure 28a, the RMSE for untrained case 1 was 0.19 at a 2% noise level and approximately 0.49 at a 5% noise level. In Figure 28b, for untrained case 2, the RMSE was 0.33 at a 2% noise level and 0.46 at a 5% noise level. In Figure 28c, for untrained case 3, the RMSE was 0.46 at a 2% noise level and 0.61 at a 5% noise level. In Figure 28d, for untrained case 4, the RMSE was 2.47 at a 2% noise level and 2.49 at a 5% noise level. Overall, reducing the data in the training dataset led to a decrease in the accuracy of the 1D CNN model.

## 6. Conclusions

In this study, we presented the 1D CNN deep regression learning of electromechanical impedance (EMI) responses from capsule-like smart aggregate (CSA) sensors embedded in a concrete structure for stress monitoring. The EMI measurement model’s theory for the CSA sensor embedded in a concrete structure and its behavior under compression were described. The 1D CNN model was developed to adapt raw CSA’s EMI responses for concrete stress estimation. The performance of the 1D CNN model in stress regression was tested under noise-contaminated EMI and untrained stress EMI cases.

Based on the analyzed results, the following conclusions can be reached:

(1) When the CSA was embedded in concrete, the peak frequency and the peak magnitude were reduced due to the effect of increasing damping and mass. In contrast with the unembedded CSA case [15], the embedded CSA case showed nonlinear changes in the RMSD and CCD metrics with concrete stress. When the concrete was damaged, there was a sudden increase in the CCD index.
(2) The 1D CNN model successfully processed and effectively extracted stress-sensitive features from raw EMI signals via the CSA sensor. The model quickly estimated concrete stress values with small errors (in the MPa unit), which makes it suitable for realistic applications requiring real-time measurement and predictive maintenance.
(3) In noise-contaminated EMI cases, the 1D CNN model’s ability to accurately estimate stress was considerably compromised by the noise effects on EMI signals. In untrained stress EMI cases, the model became less accurate, as many stress levels were omitted from the training data sets.
(4) The backbone of the 1D CNN model not only extracted stress-sensitive features well from the EMI responses of the SA sensor [24] but also efficiently processed the CSA’s EMI responses. This shows promise for practical applications which need to process multiple signal types of sensors in parallel.

Although promising results have been found, future studies are needed to

(1) investigate the behavior of the EMI spectrum with the developed CSA sensors in the evolution of concrete stiffness [13,33]; (2) investigate the effect of different moisture levels [34] on constrained CSA’s EMI responses and develop a more rigorous CNN model to minimize moisture effect; (3) develop EMI measurement methods considering the variability of EMI signals induced by temperature; (4) improve the accuracy of the 1D CNN model via optimizing modal parameters (e.g., layers, hyper-parameters, etc.); (5) integrate the current algorithm with robust classification algorithms to autonomously output multiple concrete damage status (e.g., internal damage, cracks, corrosion, etc.), assuring a more comprehensive assessment of investigated concrete structures. 

## Figures and Tables

**Table 1:** Specifications of 1D CNN layers.

No	Type	Depth	Filter	Stride	No	Type	Depth	Filter	Stride
1	Conv	4	1 × 6	1	9	Maxpool	-	1 × 2	1
2	ReLU	-	-	-	10	Conv	8	1 × 5	1
3	Maxpool	-	1 × 2	1	11	ReLU	-	-	-
4	Conv	4	1 × 4	1	12	Maxpool	-	1 × 2	1
5	ReLU	-	-	-	13	Fc1	48	-	-
6	Maxpool	-	1 × 2	1	14	Fc2	32	-	-
7	Conv	8	1 × 5	1	15	Fc3	1	-	-
8	ReLU	-	-	-	16	Regression	-	-	-

**Table 2:** Material properties of x-CSA sensor-embedded concrete cylinder.

Properties	Aluminum (6061-T6)	PZT 5A	Epoxy Layer	Concrete
Mass density, *ρ* (kg/m^3^)	2700	7750	1090	2400
Young’s modulus, *E* (GPa)	68.9	62.1	0.75	25.43
Poisson’s ratio, *ν*	0.33	0.35	0.3	0.2
Dielectric loss factor, *δ*	0.02	0.015	0.02	
Yield strength, *σ_y_* (MPa)	241			
Compressive strength, *σ_c_* (MPa)			32.3	25.3
Damping loss factor, *η*		0.0125		
Dielectric constant, ε_33_^T^ (F/m)		1.53 × 10^−8^		
Coupling constant, *d_31_* (m/V)		−1.71 × 10^−10^		

**Table 3:** EMI data labeling for stress estimation.

	Stress Level							
	S_1_	S_2_	S_3_	S_4_	S_5_	S_6_	S_7_	S_8_
Labeled stress (MPa)	2.03	4.06	6.09	8.12	10.15	12.18	14.21	15.24

**Table 4:** Data for untrained stress EMI cases.

	Scenarios			
	Untrained Case 1	Untrained Case 2	Untrained Case 3	Untrained Case 4
Training set	336	288	240	192
Validation set	7	6	5	4
Testing set	408			

## Data Availability

Data available on reasonable request from the corresponding author.
